# Supplementary material for: SynthSeg: Segmentation of brain MRI scans of any contrast and resolution without retraining
Source: Med Image Anal. Author manuscript; Available in PMC 2023 May 3. (PMC10154424; doi:10.1016/j.media.2023.102789)
Supplement: appendix A [file NIHMS1891066-supplement-appendix_A.pdf]

SynthSeg: Segmentation of brain MRI scans of any contrast and resolution without retraining
Supplementary materials

Benjamin Billot, Douglas N. Greve, Oula Puonti, Axel Thielscher, Koen Van Leemput, Bruce Fischl, Adrian V. Dalca, and Juan Eugenio Iglesias

Supplement 1: Examples of generated synthetic scans

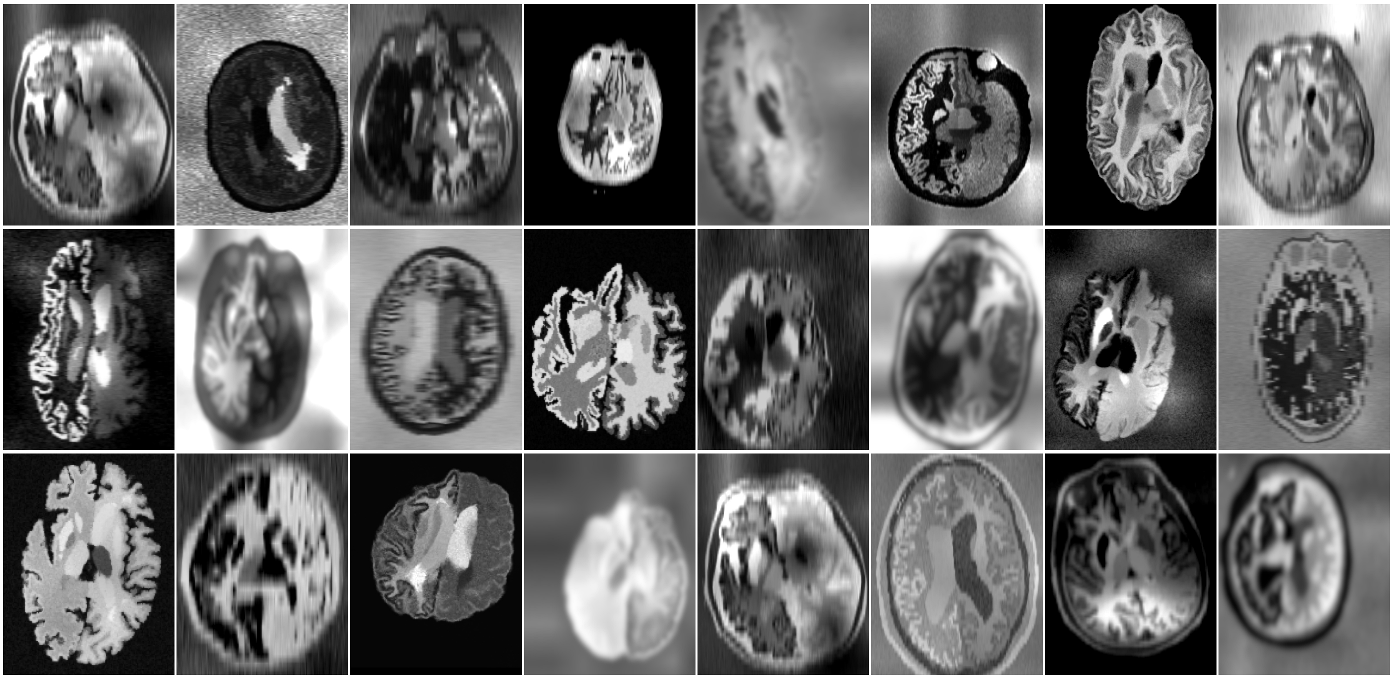

Fig. S1. Representative samples from the presented generated model. Synthetic scans present a considerable diversity in terms of contrasts and resolutions, but also in terms of sizes, shapes, bias fields, skull stripping, lesions, and anatomical morphology.

Supplement 2: Values of the generative model hyperparameters

Table S1. Values of the hyperparameters controlling the generative model. Intensity parameters assume an input in the [0, 255] interval. Rotations are expressed in degrees, and spatial measures are in millimeters.

Hyperparameter	a_{rot}	b_{rot}	a_{sc}	b_{sc}	a_{sh}	b_{sh}	a_{tr}	b_{tr}	b_{nonlin}	a_μ	b_μ	a_σ	b_σ	b_B	σ^2_γ	r_{HR}	b_{res}	a_α	b_α
Value	-20	20	0.8	1.2	-0.015	0.015	-30	30	4	0	255	0	35	0.6	0.4	1	9	0.95	1.05

Supplement 3: List of label values used during training

Table S2. List of the labels used for image synthesis, prediction, and evaluation. Note that during generation, we randomly model skull stripping with 50% probability, by removing all extra-cerebral labels from the training segmentation. In addition, we also model *imperfect* skull stripping, with a further 50% chances (so 25% of the total cases), by removing all the extra-cerebral labels except the cerebro-spinal fluid (which surrounds the brain). Different contralateral labels are used for structures marked with $^{R/L}$.

Label	removed for skull stripping simulation	predicted	evaluated
Background	N/A	yes	no
Cerebral white matter$^{R/L}$	no	yes	yes
Cerebral cortex$^{R/L}$	no	yes	yes
Lateral ventricle$^{R/L}$	no	yes	yes
Inferior Lateral Ventricle$^{R/L}$	no	yes	no
Cerebellar white matter$^{R/L}$	no	yes	yes
Cerebellar grey matter$^{R/L}$	no	yes	yes
Thalamus$^{R/L}$	no	yes	yes
Caudate$^{R/L}$	no	yes	yes
Putamen$^{R/L}$	no	yes	yes
Pallidum$^{R/L}$	no	yes	yes
Third ventricle	no	yes	yes
Fourth ventricle	no	yes	yes
Brainstem	no	yes	yes
Hippocampus$^{R/L}$	no	yes	yes
Amygdala$^{R/L}$	no	yes	yes
Accumbens area$^{R/L}$	no	yes	no
Ventral DC$^{R/L}$	no	yes	no
Cerebral vessels$^{R/L}$	no	no	no
Choroid plexus$^{R/L}$	no	no	no
White matter lesions$^{R/L}$	no	no	no
Cerebro-spinal Fluid (CSF)	yes/no	no	no
Artery	yes	no	no
Vein	yes	no	no
Eyes	yes	no	no
Optic nerve	yes	no	no
Optic chiasm	yes	no	no
Soft tissues	yes	no	no
Rectus muscles	yes	no	no
Mucosa	yes	no	no
Skin	yes	no	no
Cortical bone	yes	no	no
Cancellous bone	yes	no	no

Supplement 4: Versions of the training label maps

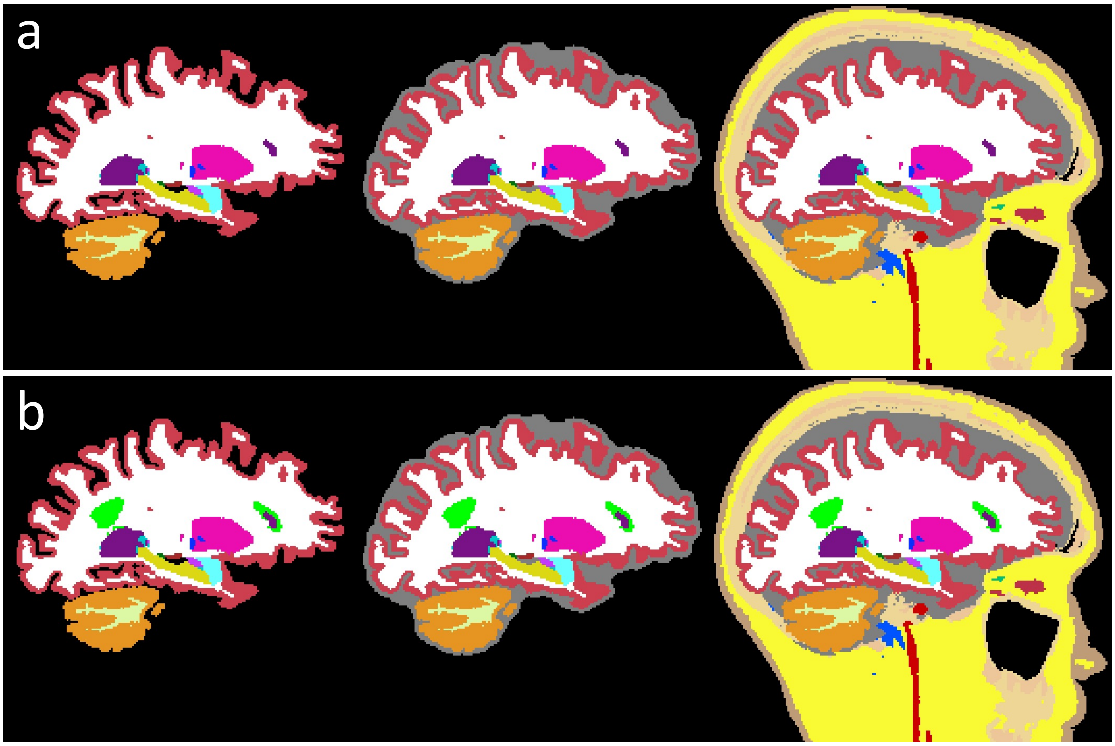

Fig. S2. Example of all versions of the label maps used during training. Each map is available (a) with, or (b) without lesion labels (bright green), and at different levels of skull stripping: perfect (left), imperfect (middle), or no skull stripping (right). Using these different versions of training label maps enables us to build robustness to white matter lesions and to (possibly imperfect) skull stripping. The lesion labels are obtained with FreeSurfer (Fischl, 2012) and directly "pasted" on the existing label maps. Training lesion labels are mainly located in the cerebral white matter, but occurrences are also found in the cerebellum, thalamus, pallidum, and putamen. Regarding the extra-cerebral labels, these are obtained with a Bayesian segmentation approach (Puonti et al., 2020).

Supplement 5: Modifications to the nnUNet, and TTA, and SIFA methods

All competing methods tested in this article are used with their default implementation, except for few minor differences that we list here. All the following modifications improve the scores obtained by the original implementations on the validation set.

nnUNet (Isensee et al., 2021)[6]: We now apply random flipping along the right/left axis (Dice score improvement of 0.09 on the validation set), as opposed to the original implementation where flipping was applied in any direction. This also mimics the augmentation strategy used for SynthSeg (see Section 4.2).

TTA (Karani et al., 2021)[7]: First, the image normaliser now uses five instead of three convolutional layers, which increases its learning capacity, especially in the case of large domain gaps (Dice improvement of 0.16 on the validation set). The second modification is relative to the training atlas that is used in Karani et al. (2021) as ground-truth during the first steps of the adaptation. Here, we add an offline step, where we rigidly register this atlas to the test scan with NiftyReg (Modat et al., 2010) We emphasise that this step was not done in the original implementation, since test scans in Karani et al. were already pre-aligned. Moreover, we also increase the number of steps during which the atlas is used by increasing the beta threshold from 0.25 to 0.4 (Karani et al., 2021) (Dice improvement of 0.06 on the validation set). Finally, we replace the existing data augmentation scheme by the same spatial, intensity and bias augmentations as for SynthSeg (Dice improvement of 0.03 on the validation set).

SIFA (Chen et al., 2019)[8]: For this method, we added an online data augmentation step during training, where we apply the same spatial, intensity and bias augmentations as for SynthSeg (Dice improvement of 0.18 on the validation set).

[6]https://github.com/MIC-DKFZ/nnUNet
[7]https://github.com/neerakara/test-time-adaptable-neural-networks-for-domain-generalization
[8]https://github.com/cchen-cc/SIFA

Supplement 6: Number of retraining for each value of N

Table S3. Number of label maps and associated retrainings used to assess performance against the amount of training subjects. Scores are then averaged across the retrainings. We emphasise that the number of retrainings is higher for low values of N to compensate for the greater variability in random subject selection. All training label maps are randomly taken from the manual segmentations of T1-39.

Number of training segmentations	1	5	10	15	20
Number of retrainings	8	5	4	3	2

Supplement 7: Training label maps for cardiac segmentation

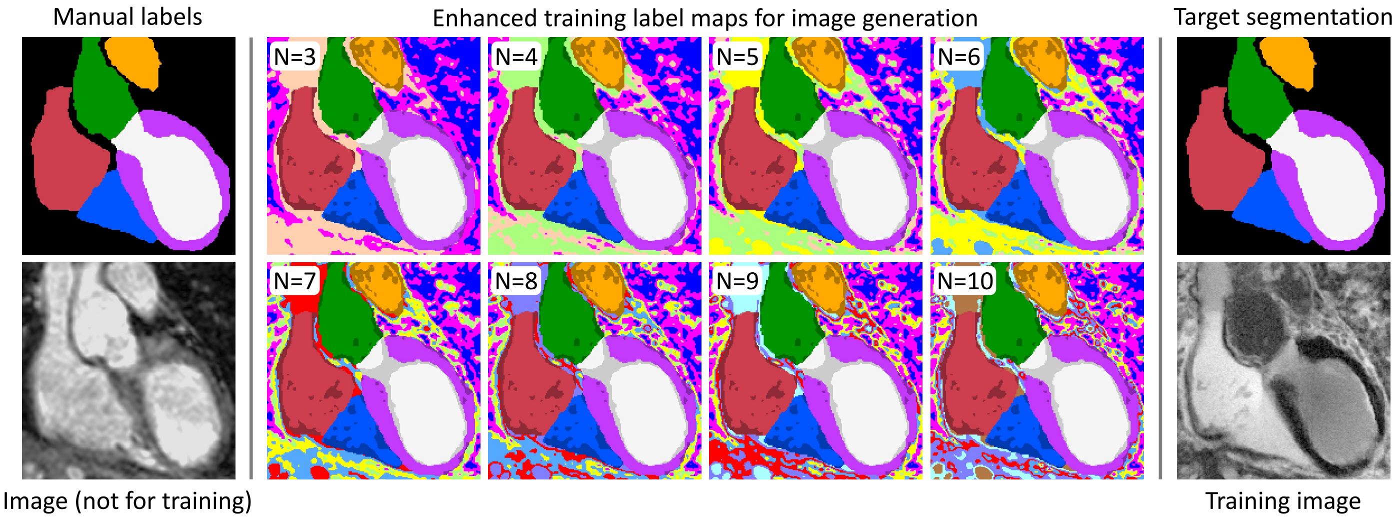

Fig. S3. Training label maps for extension to cardiac segmentation are obtained by combining three types of labels. We first start with manual delineations (top left). Second, we obtain labels for sub-regions (represented in the middle label maps by slightly shaded colours) of each of these foreground regions by clustering the associated intensities in the corresponding image (bottom left). Third, we obtain automated labels for the background structures (i.e., vessels, bronchi, bones, etc., for which no manual segmentations are available), by clustering the corresponding intensities into N classes ($N \in [3, 10]$), in order to model them with different levels of granularity. During training, one of these enhanced label maps is randomly selected to synthesise a training image (bottom right) by using the proposed generative model. Note that the target segmentation is reset to the initial manual labels.

Supplement 8: Values of the generative model hyperparameters used in the heart experiments

Table S4. Values of the hyperparameters controlling the generative model used in the heart experiments. As before, intensity parameters assume an input in the [0, 255] interval. Rotations are expressed in degrees, and spatial measures are in millimeters.

Hyperparameter	a_{rot}	b_{rot}	a_{sc}	b_{sc}	a_{sh}	b_{sh}	a_{tr}	b_{tr}	b_{nonlin}	a_{μ}	b_{μ}	a_{σ}	b_{σ}	b_B	σ^2_{γ}	r_{HR}	b_{res}	a_{α}	b_{α}
Value	-45	45	0.8	1.2	-0.02	0.02	-40	40	8	0	255	0	35	0.7	0.5	1	10	0.95	1.05
